# Dr. Alexander Marcet

**Published:** 1823-01

**Authors:** 


### OBITUARY.

---

### Dr. Alexander Marcet

Was born at Geneva in the year 1770. He gave early indications of a thirst for knowledge, and had already distinguished himself by his proficiency in the usual course of elementary studies, when his attention was suddenly turned to commerce, in consequence of the dying injunction of his father, who, himself a respectable merchant at Geneva, was anxious that his son should succeed him in the same vocation. Young Marcet was at first earnestly bent upon fulfilling the wishes of his deceased parent; but the experience of two years served but to confirm the dislike he had originally felt to a commercial life. Convinced, at length, that his repugnance was not to be overcome, he quitted for ever the dull routine of the counting-house, and yielded to the superior fascinations of literature and of science, which presented a field of inquiry so much more congenial to the natural ardour of his mind. He applied himself more particularly to the study of the law; when his views in life were again destined to be changed. The political troubles which long agitated the republic of Geneva, in the early periods of the French Revolution, defeated all his plans, and even endangered his personal safety. The faction of the day made use of the pretext that he had served as an officer in the national militia, in order to throw him into prison, from whence, in those disastrous times, there was usually so quick a transition to the scaffold. His life was fortunately saved by a change which took place in the governing party, on the death of Robespierre, and which enabled him, though with much difficulty, to obtain as a special favour the sentence of banishment for five years. On being thus obliged to quit his country, he formed the resolution of devoting himself to the study of medicine; and with this view repaired to Edinburgh, in the autumn of 1794; and, after the usual period of three years, took his degree at that university. He manifested his predilection for chemistry by selecting Diabetes as the subject of his inaugural dissertation,—a disease concerning which some very plausible chemical theories had been recently proposed, and at that time engaged considerable attention at the university.

Dr. Marcet now determined to establish himself as physician in London. He obtained the appointment of assistant physician to the Public Dispensary in Cary-street; and, in the year 1799, was elected physician to the City Dispensary. He married, about this period, the daughter of the late Mr. Haldimand, a merchant of the highest respectability in London. By a special Act of Parliament, passed in 1800, he became a naturalized subject of Great Britain.

In 1802, he was elected one of the physicians to Guy's Hospital, on the resignation of Dr. Harvey. Zealously attached to his profession, he cultivated with the greatest diligence the ample field of experience of which he thus had the command. He was in the constant habit of noting down, with great minuteness, the history and daily variations in the symptoms of every case that fell under his observation, and that presented any point of interest, both in his hospital and his private practice. The voluminous records of this nature which he has left are striking testimonials of his great and persevering industry, in the midst of his numerous other avocations. Chemistry, however, still continued to be his favourite pursuit, and he soon became eminent for the extent and correctness of his knowledge in this branch of science. He was particularly distinguished by his skill in analytical researches, and his extreme precision in the mode of conducting them. He had fitted up an excellent laboratory, which was a model of neatness and of order. He was associated for many years with Mr. William Allen, as chemical lecturer at the medical school of Guy's Hospital, and contributed in no small degree to establish its reputation in that department.

Dr. Marcet was indeed indefatigable in the promotion of every object of public utility, to which he conceived his efforts could contribute; and no person was better qualified, by the persuasive suavity of his manners, the earnestness with which he pursued what he thought was good, and the generous ardour of his disposition, to excite the zeal of others, to overcome their prejudices, and to secure their co-operation in every laudable undertaking. These qualities were eminently displayed in the establishment of the Medical and Chirurgical Society of London, an institution of which Dr. Marcet and Dr. Yelloly, in conjunction, originally conceived the plan and laid the foundations, and which has been indebted to them, more than to any other individuals, for its continued and increasing prosperity.

The services which he rendered to the medical school at Guy's Hospital, by the removal of several obstacles which formerly stood in the way of a principal source of medical knowledge, will long be remembered with gratitude by the pupils who resort to it for their education. He was also the means of effecting a reformation of a still more important nature, with regard to the diet appropriated to the patients in the hospital. He had considerable difficulty in bringing about this salutary change: it was not finally adopted, indeed, till after he had quitted his situation as physician to that establishment; but the plan of the improved system was his own, and it was entirely owing to the pains which he had taken in collecting evidence on the subject, and in strongly urging its propriety, and even necessity, that it was at length accomplished. The success of this measure was highly gratifying to him, and he always regarded it as one of the most useful things that he had ever done. He also introduced the plan of clinical lectures at Guy's Hospital, and gave several courses in conjunction with his colleagues.

The influence of his activity and public spirit extended itself to many other institutions, besides that to which he was particularly attached. We have already adverted to the leading part which he took in conducting the affairs of the Medical and Chirurgical Society; but his valuable assistance was also given to the concerns of the Royal Society, the Geological Society, the Royal Institution, and the Northern Dispensary. He was principally instrumental in bringing the Institution for the Cure and Prevention of Contagious Fevers, now known by the name of the London Fever Hospital, before the notice of Parliament, through the late Sir Samuel Romilly and the Hon. H. G. Bennet; and in thus obtaining a pecuniary grant for that useful establishment.

The following list of his contributions to various periodical Journals and Transactions of learned Societies, arranged in the order of their dates, is of itself the best evidence that can be given of his indefatigable spirit of inquiry, and of the extent of the obligations which science owes to him.

In 1799, he wrote an account of the History and Dissection of a Diabetic Case, (published in the *London Medical and Physical Journal*, vol. ii. p. 209.)

In 1801, a paper on the Medicinal Properties of the Oxyd of Bismuth, (*Memoirs of the Medical Society of London*, vol. vi. p. 155.) This paper, though read to the Society in 1801, was not published till 1805.

On the Hospice de la Maternité at Paris, (*Monthly Magazine* for May 1801, p. 311.) To this communication he did not affix his name.

In 1802, Translation of the Report to the Institute of France respecting Paul's Manufactory of Mineral Waters; with a preface written by himself. This pamphlet was published anonymously.

In 1803, a correspondence appeared between Dr. Marcet and Dr. Jenner, respecting a mode of procuring vaccine fluid, in the *London Medical and Physical Journal*, vol. ix. p. 462.

In 1805, an Analysis of the Brighton Chalybeate, published in *Dr. Saunders's Treatise on Mineral Waters*, second edition, p. 331.

Account of the Case and Dissection of a Blue Girl, in the *Edinburgh Medical Journal*, vol. i. p. 412.

In 1807, an Analysis of the Waters of the Dead Sea, and of the River Jordan, (*Philosophical Transactions* for 1807.)

In 1809, an Account of the Effects produced by a large quantity of Laudanum, taken internally, and of the means used to counteract those effects, (*Medico-Chirurgical Transactions*, vol. i. p. 77.)

A Case of Hydrophobia, with an Account of the Appearances after Death, (*Medico-Chirurgical Transactions*, vol. i. p. 132.)

In 1811, a Chemical Account of an Aluminous Chalybeate Spring in the Isle of Wight, (*Geological Transactions*, vol. i. p. 213.)

An Account of a severe Case of Erythema, not brought on by Mercury. (*Medico-Chirurgical Transactions*, vol. ii. p. 73.)

Experiments on the Appearance, in the Urine, of certain Substances taken into the Stomach, in a letter to Dr. Wollaston, (*Philosophical Trans.* for 1811, p. 106.)

A Chemical Account of various Dropsical Fluids; with Remarks concerning the Nature of the Alkaline Matter contained in these Fluids, and in the Serum of the Blood, (*Medico-Chirurgical Transactions*, vol. ii. p. 340.)

In 1812, he was engaged in a controversy with Dr. Pearson, respecting the nature of the Alkali existing in the Blood. See *Nicholson's Journal*, vol. xxxii. p. 37, and *Philosophical Magazine*, vol. xxxix.; together with a correspondence with Dr. Bostock on the same subject, (*Nicholson's Journal*, vol. xxxiii. p. 148, and 285.

In 1813, a paper on Sulphuret of Carbon, written conjointly with Professor Berzelius, (*Philosophical Transactions* for 1813, p. 171.)

On the intense Cold produced by the Evaporation of Sulphuret of Carbon, *ib.* 252.

On the Congelation of Mercury by means of Ether and the Air-pump, (*Nicholson's Journal*, vol. xxxiv. p. 119.)

Observations on Klaproth's Analysis of the Waters of the Dead Sea, (*Thomson's Annals of Philosophy*, vol. i. p. 132.)

An easy Method of procuring an intense Heat, (*ibid.* vol. ii. p. 99.)

In 1814, the articles POTASSIUM and PLATINA, in *Rees's Cyclopedia.*

Account of the Public Schools at Geneva, (*Monthly Mag.* for 1814, vol. xxxviii. p. 221 and 307.)

In 1815, some Experiments on the Chemical Nature of Chyle, with a few Observations upon Chyme, (*Medico-Chirurgical Transactions*, vol. vi. p. 618.)

In the same work there have appeared, at different times, communications from him on the subject of the employment of Nitrate of Silver as a Test of the presence of Arsenic. See vol. ii. p. 155; vol. iii. p. 342; and vol. vi. p. 663.

In 1816, Particulars respecting the Case of Professor De Saussure, (*ibid.* vol. vii. p. 228.) On the Medicinal Properties of Stramonium, with illustrative Cases, (*ibid.* vol. vii. p. 551.) And on the Preparation of the Extract, vol. vii. p. 594.

In 1817, appeared his valuable work, entitled "An Essay on the Chemical History and Treatment of Calculous Disorders;" of which a second edition was published in 1819.

In 1819, he published an Introductory Clinical Lecture.

History of a Case of Nephritis Calculosa, in which the various periods and symptoms of the disease are strikingly illustrated; and an Account of the Operation of Lithotomy, given by the patient himself, (*Med.-Chir. Trans.* vol. x. p. 147.)

On the Specific Gravity and Temperature of Sea-waters, in different parts of the Ocean, and in particular Seas; with some account of their saline contents, (*Philosophical Transactions for* 1819, p. 161.)

A paper, in French, on the subject of Vaccination, (*Bibliothèque Universelle* for November 1819.)

In 1822, Account of a singular Variety of Urine, which turned black soon after being discharged; with some particulars respecting its Chemical Properties, (*Medico-Chirurgical Transactions*, vol. xii. p. 37.)

Account of a Man who lived ten years after having swallowed a number of Clasp-knives, with a Description of the Appearances of the Body after Death, (*ibid.* vol. xii. p. 52.)

Some Experiments and Researches on the Saline Contents of Sea-water, undertaken with a view to correct and improve its chemical analysis, (*Philosophical Transactions* for 1822, p. 448.)

At the time when the Walcheren fever was committing dreadful ravages among the troops on their return from the expedition to Holland, in 1809, the want of additional medical assistance being urgently felt, Dr. Marcet volunteered his services, and was appointed to the superintendance of the general military hospital at Portsmouth; a duty which he performed with unremitting zeal, and which was interrupted only by himself becoming the subject of a similar disease. He was very severely affected, and recovered from it with great difficulty.

Having come into the possession of an ample fortune by the death of his father-in-law, he determined to retire from practice, and devote his time more exclusively to the cultivation of science. He resigned his office of physician to Guy's Hospital, but continued for a year longer to instruct the pupils in chemistry. The fortunate change which had taken place in the political state of Geneva, now restored to its independence, had induced him to revisit it, with his family, in the year 1815. During a still longer residence there in the years 1820 and 21, he felt the influence of early impressions revive with irresistible force; and the renewed ties of family and of friendship conspired, with the hope of being able materially to promote the public welfare, in rivetting his attachment to his native land. The same active spirit of philanthropy which had ever characterized his mind, displayed itself on this new field of useful exertion. Ever bent on being useful to the public, he accepted the office of member of the Representative Council of Geneva, and the appointment of honorary Professor of Chemistry at the University of that place. In conjunction with his colleague, Professor De la Riva, he gave a course of lectures on Chemistry, in the Laboratory of the Museum, in the spring of 1820.

He returned to England in the autumn of 1821, to spend the ensuing winter in London, but with the intention of afterwards transferring his whole establishment to Geneva, and of permanently fixing his abode in that country, with his lady and family, which consisted of a son and two daughters. His attachment to England, however, was still ardent, and he proposed frequently revisiting a country endeared to him by so many powerful associations. Previous to his intended removal, which was to have taken place in the autumn of this year (1822), he executed a design he had long at heart of making the tour of Scotland. This plan he accomplished to his complete satisfaction; and had returned to London in the full enjoyment of health, and with every prospect of a long continuance of happiness to himself, and to the numerous circle of relations and friends, who now mourn his loss. He was seized, while in the neighbourhood of London, with a sudden attack of gout in the stomach, from the effects of which he had scarcely recovered, when a return of the disorder took place, and was immediately fatal. He died in London on the 18th of October, at the age of fifty-two; and was interred at Battersea, near one of his sons, whom he had lost at an early age, a few years before.

The great number of objects, both public and scientific, which had thus engaged his attention, alone afford strong testimony of the active zeal with which he was animated for the advancement of knowledge and the interests of humanity. The persevering energy with which he pursued those objects, and the variety of talents and rectitude of judgment which marked his progress in whatever he undertook, are evinced by the success with which his exertions have been attended. Endeared as he was to a wide circle of friends, by the excellence of his heart, the warmth of his affections, and high sense of honour, his death has left a mournful and irreparable chasm in their society. Gifted by nature with that constitutional flow of cheerfulness which imparts the keenest relish for the enjoyments of life, he conjoined with it that expansive benevolence which seeks to render others participators in the same feelings; and it was his lot to be placed in circumstances peculiarly calculated to ensure happiness. United to a lady of congenial tastes, and of extraordinary mental accomplishments,—blessed with a family of children, —prosperous in his circumstances,—pursuing objects of interest most adapted to his talents,—enjoying a high reputation, both here and on the continent,—and living in the midst of the highly intellectual society of London, every blessing which this earth is capable of affording seemed accumulated around him. He had before him the prospect of a long career of happiness to himself, and of usefulness to his friends and to his country. The sudden dissolution of all these prospects furnishes an impressive lesson of the precarious tenure by which we hold every human good.

